# Metagenomics of Wastewater Influent from Wastewater Treatment Facilities across Ontario in the Era of Emerging SARS-CoV-2 Variants of Concern

**DOI:** 10.1128/mra.00362-22

**Published:** 2022-05-31

**Authors:** Opeyemi U. Lawal, Linkang Zhang, Valeria R. Parreira, R. Stephen Brown, Charles Chettleburgh, Nora Dannah, Robert Delatolla, Kimberly A. Gilbride, Tyson E. Graber, Golam Islam, James Knockleby, Sean Ma, Hanlan McDougall, R. Michael McKay, Aleksandra Mloszewska, Claire Oswald, Mark Servos, Megan Swinwood-Sky, Gustavo Ybazeta, Marc Habash, Lawrence Goodridge

**Affiliations:** a Canadian Research Institute for Food Safety, Department of Food Science, University of Guelph, Guelph, Ontario, Canada; b Department of Chemistry, Queen's University, Kingston, Ontario, Canada; c School of Environmental Sciences, University of Guelph, Guelph, Ontario, Canada; d Department of Chemistry and Biology, Ryerson University, Toronto, Ontario, Canada; e Department of Civil Engineering, University of Ottawa, Ottawa, Ontario, Canada; f Urban Water Research Centre, Ryerson University, Toronto, Ontario, Canada; g Children's Hospital of Eastern Ontario Research Institute, Ottawa, Ontario, Canada; h Faculty of Science, Ontario Tech University, Oshawa, Ontario, Canada; i Health Sciences North Research Institute, Sudbury, Ontario, Canada; j Great Lakes Institute for Environmental Research, University of Windsor, Windsor, Ontario, Canada; k Department of Geography and Environmental Studies, Ryerson University, Toronto, Ontario, Canada; l Department of Biology, University of Waterloo, Waterloo, Ontario, Canada; DOE Joint Genome Institute

## Abstract

We report metagenomic sequencing analyses of severe acute respiratory syndrome coronavirus 2 (SARS-CoV-2) RNA in composite wastewater influent from 10 regions in Ontario, Canada, during the transition between Delta and Omicron variants of concern. The Delta and Omicron BA.1/BA.1.1 and BA.2-defining mutations occurring in various frequencies were reported in the consensus and subconsensus sequences of the composite samples.

## ANNOUNCEMENT

The emergence and global spread of severe acute respiratory syndrome coronavirus 2 (SARS-CoV-2) (genus *Betacoronavirus*, family *Coronaviridae*) variants of concern (VOCs) pose a significant threat to global health (1, 2). SARS-CoV-2 is shed in human fecal matter and less so in urine by infected individuals, and its detection in wastewater triggered global interest in tracking the dissemination of its VOCs (3, 4). The province of Ontario and other jurisdictions have adopted genomic surveillance of wastewater for monitoring known and emerging SARS-CoV-2 VOCs in the community (5–12).

We collected 24-h composite samples of raw influent from 28 wastewater treatment plants in 10 regions across Ontario between November 2021 and February 2022 (*n* = 48) (Table 1) as part of the COVID-19 Regional Genomic Initiative (CORGI) in Ontario. Nanotrap magnetic virus particles (Ceres Nanosciences) were added to 50-mL wastewater samples to capture and concentrate the virus, followed by RNA extraction with the QIAamp viral RNA minikit (Qiagen) according to the manufacturer’s instructions. The CDC N1 and N2 regions were detected in the RNA samples with one-step reverse transcriptase quantitative PCR (RT-qPCR) performed on QuantStudio 5 (Thermo Fisher Scientific) (13) by using the 2019-nCoV CDC RUO kit (IDT, Coralville, USA) and TaqPath master mix (Thermo Fisher Scientific) as described (14). Wastewater samples with a cycle threshold of ≤35 were sequenced. For genomic sequencing, cDNA synthesis was performed using the SuperScript IV first-strand synthesis system (Thermo Fisher Scientific). SARS-CoV-2 amplicons were generated as previously described (15) but with ARTIC V4 primers (https://github.com/artic-network/artic-ncov2019/tree/master/primer_schemes/nCoV-2019). DNA libraries were generated using the Nextera XT DNA library prep kit (Illumina). Paired-end (2 × 150 bp) sequencing was performed using the MiniSeq system (Illumina). Raw sequence reads were analyzed using ViralRecon v2.4.1 (16). Variants were called with iVar v1.3.1 (17) using minimum quality and depth of 15 and 10, respectively. Consensus and subconsensus sequences were defined using mutation frequency thresholds of >50% and 10 to 50%, respectively. Variant lineages were inferred using Pangolin v3.1.20 (18). Default parameters were used for all tools unless otherwise specified.

**TABLE 1:** Summary of sequencing data of the samples

Sample ID	Date of collection (day-mo-yr)^*a*^	Sampling point	Wastewater sample location ID	Region	GPS coordinates	No. of input reads	% mapped reads	% breadth of coverage	Variant detected (consensus)	Variant detected (subconsensus)	SRA accession no.
109_2	29-Nov-21	Frontenac WWTP-1	Kingston/Ravensview WWTP	Frontenac	44.241251, −76.420616	3,859,510	80.39	99.08	Delta sublineage AY.25	None	SRR18762211
171	2-Dec-21	Durham WWTP-1	Midland/S3.2.12.21	Durham	44.75695003, −79.87483845	2,990,080	55.65	96.74	Delta sublineage AY.25.3	None	SRR18680489
115	3-Dec-21	Windsor WWTP-1	Windsor Lou Romano WRP 172	Windsor	42.28384, −83.08527	2,728,910	58.49	98.18	Delta B.1.617.2	None	SRR18680486
117	3-Dec-21	Windsor WWTP-2	Leamington PCC 148	Windsor	42.03443, −82.58850	2,794,614	43.32	98.17	Delta sublineage AY.103	None	SRR18680474
170	5-Dec-21	Durham WWTP-2	Ajax/F07.5.12.21	Durham	43.83831355, −79.0418419	2,828,286	90.16	96.66	Delta sublineage AY.121	Traces of Omicron BA.1	SRR18680490
174	5-Dec-21	Durham WWTP-3	Orillia/S4.5.12.21	Durham	44.59004157, −79.4132297	2,917,708	96.19	96.47	Delta sublineage AY.25.3	None	SRR18680488
116	6-Dec-21	Windsor WWTP-1	Windsor Lou Romano WRP 173	Windsor	42.28384, −83.08527	2,649,640	54.05	90.05	Delta sublineage AY.4	None	SRR18680485
118	6-Dec-21	Windsor WWTP-2	Leamington PCC 149	Windsor	42.03443, −82.58850	3,013,594	44.39	97.40	Delta sublineage AY.103	None	SRR18680463
150	8-Dec-21	Northern Ontario WWTP-1	Sudbury/TPKL221208	Northern Ontario	46.4655660272402, −81.0328557166295	2,145,830	30.57	98.75	Delta sublineage AY.74	None	SRR18680452
155	9-Dec-21	Toronto WWTP-1-Site 1	S2A-177	Toronto	43.73584, −79.495832	2,767,236	52.66	96.52	Delta B.1.617.2	Traces of Omicron BA.1	SRR18680491
176	13-Dec-21	Windsor WWTP-2	Leamington PCC 152	Windsor	42.03443, −82.58850	2,837,338	96.14	97.52	Delta sublineage AY.103	None	SRR18680487
284	15-Dec-21	Halton WWTP-1	Maplehurst Correctional Complex	Halton	43.520000, −79.900000	3,446,194	99.64	99.14	Delta sublineage AY.25.1	None	SRR18680462
210	17-Dec-21	Guelph WWTP-1	Guelph	Wellington	43.520000, −80.270000	2,458,380	75.02	96.74	Delta sublineage AY.39	Omicron BA.1	SRR18680480
208	20-Dec-21	Guelph WWTP-1	Guelph	Wellington	43.520000, −80.270000	3,406,436	67.05	98.86	Omicron BA.1	Delta B.1.617.2	SRR18680481
191	21-Dec-21	Frontenac WWTP-2	Loyalist/Amherstview WPCP	Frontenac	44.233127, −76.660988	3,231,114	49.13	98.79	Omicron BA.1	Delta B.1.617.2	SRR18680484
200	21-Dec-21	Niagara WWTP-1	Crystal Beach Wastewater Treatment Plant (Fort Erie)	Niagara	42.860000, −79.060000	3,075,436	35.10	99.69	Omicron BA.1.1	Delta B.1.617.2	SRR18680482
211	22-Dec-21	Guelph WWTP-1	Guelph	Wellington	43.520000, −80.270000	3,053,446	80.99	98.71	Omicron BA.1.1	Delta B.1.617.2	SRR18680479
192	23-Dec-21	Frontenac WWTP-3	Eastern Ontario/Hawkesbury	Frontenac	45.611676, −74.596091	4,867,030	96.39	98.12	Omicron BA.1.1	Delta B.1.617.2	SRR18680483
287	29-Dec-21	Halton WWTP-1	Maplehurst Correctional Complex	Halton	43.520000, −79.900000	3,058,088	91.51	99.08	Delta sublineage AY.25.1	Traces of Omicron BA.1	SRR18680461
250	3-Jan-22	Frontenac WWTP-4	Kingston/Cataraqui Bay WWTP	Frontenac	44.214819, −76.550722	3,616,518	82.89	98.34	Omicron BA.1.1	Delta B.1.617.2, Traces of Omicron BA.2	SRR18680467
304	3-Jan-22	Niagara WWTP-2	Queenston Wastewater Treatment Plant	Niagara	43.160000, −79.050000	3,891,574	76.68	98.88	Omicron BA.1.1	Delta B.1.617.2	SRR18680460
222	4-Jan-22	Durham WWTP-4	Courtice WWTP/C04.4.1.22	Durham	43.87131928, −78.7559778	3,276,026	68.05	98.29	Omicron BA.1.1	Delta B.1.617.2	SRR18680478
227	5-Jan-22	Toronto WWTP-1-Site 2	S2B-167	Toronto	43.76187, −79.508388	3,884,772	98.55	97.02	Omicron BA.1.1	None	SRR18680477
229	5-Jan-22	Toronto WWTP-1-Site 3	S1B-190	Toronto	43.730131, −79.549578	3,068,436	73.87	97.54	Omicron BA.1.1	None	SRR18680476
251	5-Jan-22	Frontenac WWTP-5	Eastern Ontario/Hawkesbury WWTP	Frontenac	45.611676, −74.596091	3,373,878	67.69	98.40	Omicron BA.1	Delta B.1.617.2	SRR18680466
230	6-Jan-22	Waterloo WWTP-1	Waterloo 845R-I-B 1/6	Waterloo	43.48509, −80.50412	2,655,472	97.84	99.68	Omicron BA.1.1	Delta B.1.617.2	SRR18680475
231	6-Jan-22	Waterloo WWTP-2	Galt 847R-I-B 1/6	Waterloo	43.340827, −80.313357	3,138,934	98.75	99.59	Omicron BA.1.1	Delta B.1.617.2	SRR18680473
232	6-Jan-22	Waterloo WWTP-3	Kitchener 846R-I-B 1/6	Waterloo	43.396587, −80.421080	3,486,778	88.36	99.71	Omicron BA.1.1	Delta B.1.617.2	SRR18680472
233	7-Jan-22	Waterloo WWTP-4	Peel 830F-I-B 1/7	Waterloo	43.7546220, −79.6264007	2,987,086	53.29	99.57	Omicron BA.1.1	Delta B.1.617.2	SRR18680471
235	7-Jan-22	Waterloo WWTP-5	York 831F-I-B 1/7	Waterloo	43.8456555, −79.3350315	2,720,060	76.96	98.40	Omicron BA.1.1	None	SRR18680470
254	10-Jan-22	Northern Ontario WWTP-1	Sudbury/TPKL220110	Northern Ontario	46.4655660272402, −81.0328557166295	3,003,880	15.27	99.69	Omicron BA.1.1	Delta B.1.617.2	SRR18680465
307	10-Jan-22	Guelph WWTP-1	Guelph	Wellington	43.520000, −80.270000	3,532,702	99.75	97.55	Omicron BA.1.1	Delta B.1.617.2	SRR18680459
245	11-Jan-22	Toronto WWTP-1-Site 1	S2A-187	Toronto	43.73584, −79.495832	2,095,676	98.73	99.77	Omicron BA.1.1	Delta B.1.617.2	SRR18680469
368	12-Jan-22	Niagara WWTP-3	Port Weller Sewer Treatment Plant	Niagara	43.230000, −79.220000	3,412,742	94.39	100.00	Omicron BA.1.1	Delta B.1.617.2	SRR18680450
248	13-Jan-22	Toronto WWTP-1-Site 1	S2A-189	Toronto	43.73584, −79.495832	3,149,968	91.69	98.63	Omicron BA.1.1	Delta B.1.617.2	SRR18680468
365	13-Jan-22	Niagara WWTP-4	Baker Road Wastewater Treatment Plant (Grimsby)	Niagara	43.190000, −79.540000	2,374,706	98.36	99.00	Omicron BA.1.1	Delta B.1.617.2	SRR18680451
330	14-Jan-22	Halton WWTP-1	Maplehurst Correctional Complex	Halton	43.520000, −79.900000	3,637,030	97.02	99.69	Omicron BA.1.1	Delta B.1.617.2	SRR18680458
341	14-Jan-22	Guelph WWTP-1	Guelph	Wellington	43.520000, −80.270000	3,529,346	74.85	97.64	Omicron BA.1.1	Delta B.1.617.2	SRR18680454
257	17-Jan-22	Northern Ontario WWTP-1	Sudbury/TPKL220117	Northern Ontario	46.4655660272402, −81.0328557166295	3,175,686	91.77	99.70	Omicron BA.1.1	Delta B.1.617.2	SRR18680464
373	17-Jan-22	Northern Ontario WWTP-2	Sault Ste. Marie/TPSSM220117	Northern Ontario	46.5057224005555, −84.25739500029	3,313,904	89.77	99.00	Omicron BA.1.1	Delta B.1.617.2, Omicron BA.2	SRR18680448
338	18-Jan-22	Niagara WWTP-5	Niagara Falls-Stamford Wastewater Treatment Plant	Niagara	43.120000, −79.090000	2,917,972	96.83	99.71	Omicron BA.1.1	Delta B.1.617.2	SRR18680455
359	18-Jan-22	Durham WWTP-5	Corbett WWTP/OUT/A02.18.1.22	Durham	43.85542682, −78.89248301	2,366,054	85.02	99.00	Omicron BA.1.1	Delta B.1.617.2	SRR18680453
332	19-Jan-22	Halton WWTP-1	Maplehurst Correctional Complex	Halton	43.520000, −79.900000	3,260,302	40.88	99.66	Omicron BA.1.1	Delta B.1.617.2	SRR18680457
333	21-Jan-22	Halton WWTP-1	Maplehurst Correctional Complex	Halton	43.520000, −79.900000	3,834,210	99.53	99.21	Omicron BA.1.1	Delta B.1.617.2	SRR18680456
372	26-Jan-22	Northern Ontario WWTP-1	Sudbury/TPKL220126	Northern Ontario	46.4655660272402, −81.0328557166295	2,985,948	99.15	100.00	Omicron BA.1.1	Delta B.1.617.2	SRR18680449
421	5-Feb-22	Ottawa WWTP-1	Ottawa influent	Ottawa	45.461111, −75.589167	2,634,062	93.99	96.00	Omicron BA.1.1	None	SRR18680447
422	7-Feb-22	Ottawa WWTP-1	Ottawa influent	Ottawa	45.461111, −75.589167	2,482,608	99.95	96.00	Omicron BA.1.1	None	SRR18680446
423	9-Feb-22	Ottawa WWTP-1	Ottawa influent	Ottawa	45.461111, −75.589167	2,668,098	50.47	95.00	Omicron BA.1.1	None	SRR18680492

a Data in the table are sorted by date of collection. WWTP, wastewater treatment plant.

**FIG 1:**
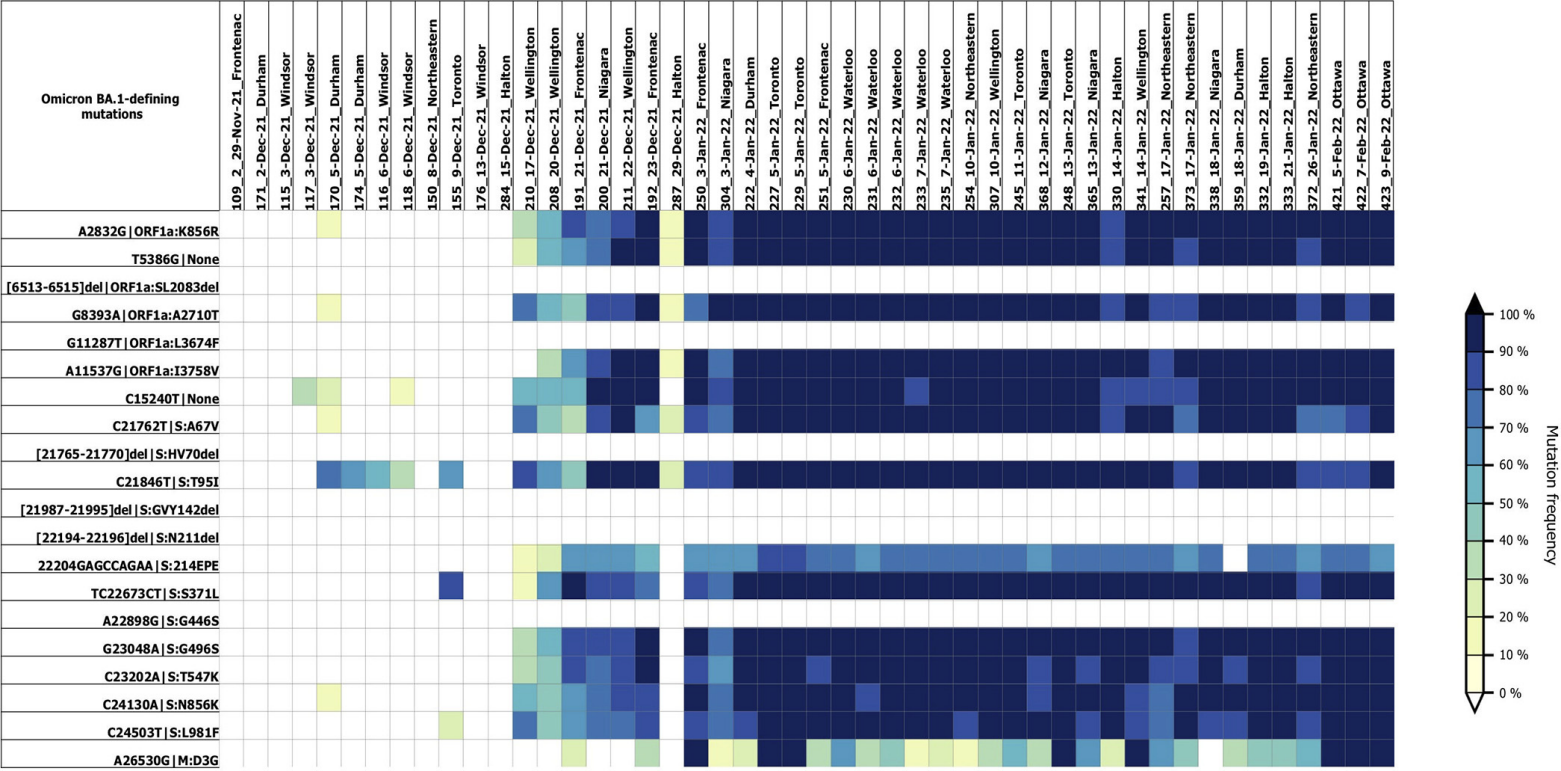
Heatmap showing estimated frequencies of Omicron (BA.1) mutations in composite samples of wastewater influent from 10 regions across Ontario. Each column represents a sample, and they are ordered by date of collection. Each row represents the genomic locus of a BA.1-defining mutation (https://github.com/cov-lineages/constellations/tree/main/constellations/definitions). Colors depict the percentage frequency of mutations in the samples; the deeper the color, the higher the mutation frequency. The heatmap was generated using VCFparser v1.0.0 (https://github.com/kbessonov1984/VCFParser).

We received 148,603,298 total reads across all 48 samples (mean, 3,095,902; range, 2,095,676 to 4,867,030). In most of the samples (85%; *n* = 41), ≥50% of reads mapped to the SARS-CoV-2 Wuhan strain. The average breadth of coverage of the consensus sequences generated was 98%. In consensus sequences, the Delta variants, including 8 sublineages, were detected in 14 samples from 7 regions in December 2021 (Table 1). Omicron BA.1/BA.1.1 was detected in 34 samples collected from all the regions studied except in Windsor, located in the extreme southwest of the province (Fig. 1). In subconsensus sequences, 26 samples contained the Delta VOC, 2 samples contained both Delta and Omicron BA.2 mutations, while traces (≤6 mutations) of Omicron BA.1-defining mutations were detected in 4 samples. Overall, multiple SARS-CoV-2 VOCs were detected in 32 samples (Table 1). Collectively, continuous genomic surveillance of wastewater provides sufficient specificity to infer individual VOC lineages in mixed samples and is effective for monitoring SARS-CoV-2 VOCs in the community.

### Data availability.

The metagenomic sequences are available in the NCBI Sequence Read Archive under BioProject accession number PRJNA824537.
